# Dynamics of computational waveform: A study of bifurcation, chaos, and sensitivity analysis

**DOI:** 10.1371/journal.pone.0326230

**Published:** 2025-07-09

**Authors:** Nur Hasan Mahmud Shahen, Md. Al Amin, Foyjonnesa, M. M. Rahman

**Affiliations:** 1 Department of Mathematics, Bangladesh University of Engineering and Technology, Dhaka, Bangladesh; 2 Department of Arts and Sciences, Bangladesh Army University of Science and Technology, Saidpur, Bangladesh; 3 Department of Mathematics, University of Rajshahi, Rajshahi, Bangladesh; 4 Department of Electrical and Electronic Engineering, Northern University Bangladesh, Dhaka, Bangladesh; Tel Aviv University, ISRAEL

## Abstract

This study delves into the extraction of solutions for the fractional-order that describes wave circulation in space-time fractional low-pass electrical transmission (LPET) lines and Drinfel’d–Sokolov–Wilson (DSW) equations. Leveraging the Sardar-subequation scheme, by applying a simple linear fractional transformation, the model equations are transformed into an ordinary differential equation. The use of the Sardar-subequation technique produces a diverse range of traveling waveform for the governing equations. The behavior of the dynamics of select solutions, representing singular and multiple soliton, kink and periodic kink, W-shaped bright soliton, dark-kink soliton and kink-like soliton solutions, is then visually showcased through their two and three-dimensional profiles with the help of computational software Maple and MATLAB. In addition, the dynamical model of the proposed DSW equation is constructed by utilizing the Galilean transformation in order to accomplish our objective. Then, using the concepts of the planar dynamical system, bifurcation, chaos, and sensitivity studies of the aforementioned model are carried out. For the aforementioned model, we find chaotic, quasi-periodic, and periodic behaviors. This research is novel in that it provides new insights into the complex dynamics of the governing model and the variety of waveforms it produces through a comprehensive investigation. By integrating waveform characterization, chaotic behavior, and bifurcation analysis, this study enhances our understanding of the nonlinear behavior of waves in shallow water.

## 1. Introduction

There has been a growing emphasis on non-linear evolution equations (NLEEs) in recent times, affecting a diverse array of fields like mathematical biology, chemical physics, optical fiber technology, mechanics, and hydrodynamics [1–4]. These equations serve as indispensable tools for capturing and describing intricate phenomena, contributing significantly to the advancement of knowledge and technological applications [5,6]. Understanding the behavior of systems governed by NLEEs has thus emerged as a fundamental pursuit, necessitating a thorough investigation into the associated wave structures [7, 8]. The exploration of wave structures in NLEEs is a critical endeavor, providing valuable discernments into the underlying dynamics of complex phenomena observed in realms such as robust state physics, fluid mechanics, atoms, physical compounds, and optical fibers [9,10]. The search for wave solutions in the NLEEs has been thought fundamental to unraveling the richness of nonlinear physical behaviors and central to the progress of scientific inquiry [11]. In response to this imperative, numerous researchers and scientists have devised efficient techniques for uncovering analytic solutions to non-linear partial differential equations, such as the adapted Kudryashov technique [12], updated simple equation approach [13], expansion of sine-Gordon equation procedure [14], enhanced $\left(\frac{G^{\prime }}{G^{2}}\right)$-expansion method [15], the extended sinh-Gordon equation technique [16], trial solution method [17], the $\left(\frac{G^{\prime }}{G},\frac{1}{G}\right)$-expansion process [18], exp(−ϕ(ξ) approach [19], multiple simplest equation technique [20], solitary wave ansatzes [21], technique of Frobenius integrable putrefaction [22], reformed extended tanh-function method [23–24], auto-Bäcklund transformation [25], the unified method [26], tanh−coth technique [27], modified Khater approach [28], trigonometric quantic B-spline strategy [29], generalized exponential rational function technique [30], advanced exp(−ϕ(ξ))-expansion process [31,32], modified direct algebraic method [33], Adomian Decomposition technique [34], sub-equation technique [35], transform technique of q-homotopy analysis [36], simplified Hirota approach [37], F-expansion technique [38], and so forth.

The nonlinear space-time fractional LPET line equation serves as a vital tool in physics [39], offering insights into the behavior of nonlinear excitations within nonlinear media along with the dynamics of novel exotic systems. Systematic and mathematical efforts have been directed towards the exploration of nonlinear excitation's using the LPETs, with the experimental confirmation of solitons in such systems [40]. The versatility of LPETs has recently been demonstrated in their efficacy for extremely wide-band signal directing and shaping, covering frequencies from dc to 100 GHz [40]. In the realm of physics, LPETs have proven to be valuable instruments for comprehending the transmission of electrical solitons, acting as voltage waves on nonlinear dispersive exteriors [41]. The mathematical model of LPETs and the derivation of exact and explicit solutions find wide-ranging applications in electronic engineering and communication engineering. These applications encompass antenna networks for television signal circulation, radio receivers and transmitters, call directing in stem lines between telephone switching centers, mobile network designs, computer network connections, high speed computer buses, and additional uses [42].

The DSW equation, introduced by Wilson [43], models dispersive water waves mathematically. This equation has had a significant impact on fluid dynamics, physics, ocean engineering, and various technical studies. The mathematical form of the DSW equation is described as follows [44]


$$\begin{array}{c} \left\{ \begin{array}{c} \;u_{t}+av_{x}v=0\\ v_{t}+buv_{x}+cu_{x}v+dv_{xxx}=0. \end{array}\right. \end{array}$$ (1)

where a,b,c, as well d are constants that are not zero.

Various integration schemes, such as $\left(\frac{G^{\prime }}{G^{2}}\right)$-expansion technique [45], adapted simple equation technique [46], unified technique [39], improved mathematical technique [47], new extended algebraic method [48], the modified Kudryashov approach [49], extended sinh-Gordon equation expansion approach [49], among others, can be found in the literature of LPET lines. On the other hand, the φ6-model expansion method [44], the modified rational expansion approach [50], the homotopy analysis method [51], the Jacobi elliptical function technique [52], the exp(−ϕ(ξ) approach [18], the Sine-Gordon expansion method [53], among others, can be found in the literature of DSW equation. These techniques aim to generate exact solitary wave structures for the LPETs and DSW model, showcasing the diverse approaches available for understanding and analyzing its nonlinear dynamics.

We have sufficient grounds to assert that the solutions presented in this study have not been previously explored. To the best of our knowledge, no prior research has applied the Sardar sub-equation method [54] to the fractional LPET and DSW models. This study aims to address this gap by investigating the effects of fractional and permissible parameters and by conducting a comparative analysis of solutions derived using various types of fractional derivatives. Furthermore, we examine the given equations using conformable derivatives, a framework that offers a meaningful and interpret able mathematical formulation.

A valuable technique for studying dynamic systems, bifurcation analysis has important applications in a variety of fields [55–57]. In 2002, Liu and Li [58] familiarized the bifurcation technique as a strong tool for exploring the dynamic characteristics of partial differential equations. Finding precise traveling wave solutions and analyzing bifurcation events are two areas in which it excels. It analyzes how variations in system parameters influence its qualitative behavior [59]. Researchers can better grasp how systems change from stable to unstable states or from chaotic behaviour thanks to this approach. Examining bifurcation analysis and novel waveform inside the first fractional LPET and DSW nonlinear framework is the principal focus of this paper. Within conformable fractional models, there are functions that can be expressed through Taylor power sequences, which are not always available in traditional calculus under certain conditions.

The conformable derivative exhibits notable proficiency when applying chain and product laws, although fractional calculus often proves more suitable for intricate designs. It’s noteworthy that the conformable derivative applied to a constant function yields zero, a contrast to cases where Riemann fractional calculus might not hold as much significance. Mittag-Leffler functions, crucial in simplifying exponential functions within fractional calculus, also warrant attention.

This study employs the LPET and DSW model within the framework of conformable derivatives, with the aim of assessing the behavior of these models in light of these observations. Through this investigation, we endeavor to offer valued intuitions into the application of the Sardar-subequation to fractional LPET and DSW models, and to illuminate the distinctive attributes provided by conformable fractional derivatives in this particular context. Respite of the part of this article is decorated as follows.

## 2. Prologs and approaches

### 2.1 Elucidation and some structures of conformable derivative (CD)

The concept of CD, as developed by Khalil et al. [60], is primarily based on the logical framework of limits.

**Definition** If we take into account a mapping f:(0,∞rightarrowℜ, then the CD of f order β can be expressed as $T_{t}^{\beta }f(t)=\lim_{\varepsilon \to 0}\left(\frac{f\left(t+\varepsilon t^{1-\beta }\right)-f(t)}{\varepsilon }\right),$ for all t>0,0<ω≤1.

Abdeljawad [61], a prominent researcher, has laid the groundwork for the use of exponential functions, Grönwall’s inequality, the chain rule, definite and indefinite integration by slices, Fourier transformation, Laplace transform, and Taylor’s series expansions in the context of CD with fractional-order progression. The description of CD may easily overpower the use of the current enhanced Riemann Liouville derivative explanation. By applying the notion of CD, the problems with the present modified Riemann-Liouville derivative definition may be successfully resolved.

**Theorem 1** Let us consider β∈(0,1], and the function f=f(t),and g=g(t), be β -CD at the point t>0, then we can write as

(i) $T_{t}^{\beta }\left(dg+cf\right)=dT_{t}^{\beta }g+cT_{t}^{\beta }f,$ for all c,d∈ℜ
(ii) $T_{t}^{\beta }\left(t\gamma \right)=\gamma \;t^{\gamma -\beta },\;$ for all γ∈ℜ
(iii)
$\begin{array}{c} T_{t}^{\beta }\left(fg\right)=gT_{t}^{\beta }\left(f\right)+f\;T_{t}^{\beta }\left(g\right)\;,\; \end{array}$

(iv)
$T_{t}^{\beta }\left(\frac{f}{g}\right)=\frac{gT_{t}^{\beta }\left(f\right)-fT_{t}^{\beta }\left(g\right)}{g^{2}}.$

Furthermore, if this function f is derivable, then 

**Theorem 2** Consider f:(0,βrightarrowR, be a real type function such that f is differentiable and β -conformable derivable. Also, assume that g be a derivable function well-specified in the domain of f. Then we have $T_{t}^{\beta }(fog,\left(t\right)=t^{1-\beta }g(t{)}^{\beta -1}g^{\prime }(t)T_{t}^{\beta }{\left(f(t)\right)}_{t=g(t)},$ where prime indicates the simple derivatives concerning tothe point t.

We took special care in our research when addressing the preferred equation and the concept of conformable derivative. Several functions do not extend Taylor’s series expansions at specific points in fundamental calculus, but they do fit within the framework of conformable order derivatives. Although complex approaches arise in the logic of fundamental fractional geometry, CD operates effectively in the product and chain rules. When the Riemann derivative of fractional order is not considered, the CD of a constant function is zero. In fractional order calculus, Mittag-Leffler functions are recognized as crucial, offering an interpretation analogous to exponential functions. For instance, within the framework of CD, the fractional order exponential function given by f(t) = e$\frac{t^{\alpha }}{\alpha },$ is particularly relevant.

### 2.2. An overview of the Sardar-subequation technique

Let us contemplate the PDE


$$Z\left(v,v_{t},v_{x},v_{tt},v_{xx},\ldots \right)=0,$$ (2)

where v(x,t) is an unfamiliar function.


$$\text{Let }v\left(x,t\right)=U\left(\xi \right),\xi =l\frac{x^{\gamma }}{\gamma }+c\frac{t^{\gamma }}{\gamma },$$ (3)

where c is a non-zero constant.

The transformation outlined above results in the derivation of the following ODE


$$T\left(U,cU^{\prime },lU^{\prime },c^{2}U^{\prime \prime },l^{2}U^{\prime \prime },\ldots \right)=0,$$ (4)

in which U=U(ξ), $U^{\prime }=\frac{dU}{d\xi },$ $U^{\prime \prime }=\frac{d^{2}U}{d\xi^{2}},$ …,.

Consider Eq. (4) has the solution as follows


$$U\left(\xi \right)=\sum_{i=0}^{N}S_{i}\mathrm{\backslash Upphi}{\left(\xi \right)}^{i},$$ (5)

where $S_{i}(0\leq i\leq N)$ are constant with $S_{N}\neq 0$ and \Upphi(ξ) is satisfied the following relation:


$$\mathrm{\backslash Upphi}\prime \left(\xi \right)=\sqrt{D+E\mathrm{\backslash Upphi}{\left(\xi \right)}^{2}+\mathrm{\backslash Upphi}{\left(\xi \right)}^{4}},$$ (6)

where D and E are constant of real type and Eq. (6) satisfied the following solution

**Case 1:** (When E>0 and D=0)


$$\begin{array}{c} {\mathrm{\backslash Upphi}}_{1}^{\pm }\left(\xi \right)=\pm \sqrt{-Eqr}\;{\text{sech}}_{qr}\left(\sqrt{E}\xi \right), \end{array}$$

$$\begin{array}{c} \varphi_{2}^{\pm }\left(\xi \right)=\pm \sqrt{Eqr}\;{\text{csch}}_{qr}\left(\sqrt{E}\xi \right), \end{array}$$

where $sech_{qr}(\xi )=\frac{2}{qe^{\xi }+re^{-\xi }}$, $csch_{qr}(\xi )=\frac{2}{qe^{\xi }-re^{-\xi }}$.

**Case 2:** (When E<0 and D=0)


$$\begin{array}{c} {\mathrm{\backslash Upphi}}_{3}^{\pm }\left(\xi \right)=\pm \sqrt{-Eqr}\;{\text{sech}}_{qr}\left(\sqrt{-E}\xi \right), \end{array}$$

$$\begin{array}{c} \varphi_{4}^{\pm }\left(\xi \right)=\pm \sqrt{-Eqr}\;{\text{csch}}_{qr}\left(\sqrt{-E}\xi \right), \end{array}$$

where $\sec_{qr}(\xi )=\frac{2}{qe^{i\xi }+re^{-i\xi }}$, $\csc_{qr}(\xi )=\frac{2i}{qe^{i\xi }-re^{-i\xi }}$.

**Case 3:** (When E<0 and $D=\frac{E^{2}}{4}$)


$${\mathrm{\backslash Upphi}}_{5}^{\pm }\left(\xi \right)=\pm \sqrt{-\frac{E}{2}}\tanh_{qr}\left(\sqrt{-\frac{E}{2}}\xi \right),$$

$$\varphi_{6}^{\pm }\left(\xi \right)=\pm \sqrt{-\frac{E}{2}}\coth_{qr}\left(\sqrt{-\frac{E}{2}}\xi \right),$$

$$\begin{array}{c} {\mathrm{\backslash Upphi}}_{7}^{\pm }\left(\xi \right)=\pm \sqrt{-\frac{E}{2}}(\tanh_{qr}\left(\sqrt{-2E}\xi \right)\pm i\sqrt{-Eqr}\;{\text{sech}}_{qr}\left(\sqrt{-2E}\xi \right)), \end{array}$$

$$\begin{array}{c} {\mathrm{\backslash Upphi}}_{8}^{\pm }\left(\xi \right)=\pm \sqrt{-\frac{E}{2}}(\coth_{qr}\left(\sqrt{-2E}\xi \right)\pm \sqrt{-Eqr}\;{\text{csch}}_{qr}\left(\sqrt{-2E}\xi \right)), \end{array}$$

$$\begin{array}{c} {\mathrm{\backslash Upphi}}_{9}^{\pm }\left(\xi \right)=\pm \sqrt{-\frac{E}{8}}(\tanh_{qr}\left(\sqrt{-\frac{E}{8}}\xi \right)\pm \sqrt{-Eqr}\;\coth_{qr}\left(\sqrt{-\frac{E}{8}}\xi \right)), \end{array}$$

where $\tanh_{qr}(\xi )=\frac{qe^{\xi }-re^{-\xi }}{qe^{\xi }+re^{-\xi }}$, $\coth_{qr}(\xi )=\frac{qe^{\xi }+re^{-\xi }}{qe^{\xi }-re^{-\xi }}$.

**Case 4:** (When E>0 and $D=\frac{E^{2}}{4}$)


$${\mathrm{\backslash Upphi}}_{10}^{\pm }\left(\xi \right)=\pm \sqrt{\frac{E}{2}}\tan_{qr}\left(\sqrt{\frac{E}{2}}\xi \right),$$

$$\begin{array}{c} \phi_{11}^{\pm }\left(\xi \right)=\pm \sqrt{\frac{E}{2}}\cot_{qr}\left(\sqrt{\frac{E}{2}}\xi \right), \end{array}$$

$${\mathrm{\backslash Upphi}}_{12}^{\pm }\left(\xi \right)=\pm \sqrt{\frac{E}{2}}(\tan_{qr}\left(\sqrt{2E}\xi \right)\pm \sqrt{Eqr}\sec_{qr}\left(\sqrt{2E}\xi \right)),$$

$${\mathrm{\backslash Upphi}}_{13}^{\pm }\left(\xi \right)=\pm \sqrt{\frac{E}{2}}(\cot_{qr}\left(\sqrt{2E}\xi \right)\pm \sqrt{Eqr}\csc_{qr}\left(\sqrt{2E}\xi \right)),$$

$${\mathrm{\backslash Upphi}}_{14}^{\pm }\left(\xi \right)=\pm \sqrt{\frac{E}{8}}(\tan_{qr}\left(\sqrt{\frac{E}{8}}\xi \right)\pm \sqrt{Eqr}\cot_{qr}\left(\sqrt{\frac{E}{8}}\xi \right)),$$

where $\tan_{qr}\left(\xi \right)=-i\frac{qe^{i\xi }-re^{-i\xi }}{qe^{i\xi }+re^{-i\xi }}$, $\cot_{qr}(\xi )=i\frac{qe^{i\xi }+re^{-i\xi }}{qe^{i\xi }-re^{-i\xi }}$.

Begin by applying the balancing rule to find the variable N in Eq. (4). Once N is determined, substitute Eq. (5) and (6) into Eq. (4) to obtain the equation expressed in terms of the power series \Upphi(ξ). Once a non-zero solution is obtained, set all coefficients of \Upphi(ξ) equal to zero, resulting in a system of algebraic equations (SAEs). This structure provides the solutions when it is solved. When these solutions are inserted, along with those from Eq. (6), we arrive at the final solution from Eq. 5.

## 3. Solicitation of space-time fractional LPET equation in Sardar-subequation method

This section employs the Sardar-subequation technique to tackle the space–time fractional regulating wave form in LPET lines. To paradigm explicit solutions of the space-time LPET stripe equation of fractional order using the Sardar-subequation method, we begin with the LPET line model of fractional order, which originates from the traditional integer-order equation as described in [39], expressed as


$$MB_{0}T_{tt}^{2\gamma }u\left(x,t\right)-T_{xx}^{2\gamma }u\left(x,t\right)-\frac{\delta^{2}}{12}T_{xxxx}^{4\gamma }u\left(x,t\right)-MB_{0}vT_{tt}^{2\gamma }u^{2}\left(x,t\right)+MB_{0}\beta T_{tt}^{2\gamma }u^{3}\left(x,t\right)=0$$

To address our proposed method, we employ wave transformation as u(x,t)=U(ξ), where $\xi =l\frac{x^{\gamma }}{\gamma }+c\frac{t^{\gamma }}{\gamma }$. The following ODE is obtained by integrating the consequential equation two time with respect to ξ and putting the constants equal to zero:


$$\left(MB_{0}c^{2}-l^{2}\right)U-MB_{0}vc^{2}U^{2}+MB_{0}\beta c^{2}uU^{3}-\frac{1}{12}\delta^{2}l^{4}U^{\prime \prime }=0$$ (7)

Implementing the homogeneous balancing concept in Eq. (7), we determine the value of N to be 1. Thus, Eq. (5) is given as follows:


$$U\left(\xi \right)=S_{0}+S_{1}\mathrm{\backslash Upphi}\left(\xi \right),$$ (8)

where $S_{0}andS_{1}$ denote real parameters that are currently unfamiliar and will be resulted subsequently.

Substituting Eq. (8) into Eq. (7), Upon making all the coefficients of U(ξ) equivalent to zero, we attain the succeeding SAEs


$$MB_{0}\beta c^{2}S_{1}^{3}-\frac{1}{6}\delta^{2}l^{4}S_{1}=0,$$

$$3M\beta c^{2}B_{0}S_{0}S_{1}^{2}-Mc^{2}vB_{0}S_{1}^{2}=0,$$

$$S_{1}Mc^{2}B_{0}-2MB_{0}vc^{2}S_{0}S_{1}-S_{1}l^{2}+3MB_{0}\beta c^{2}S_{0}^{2}S_{1}-\frac{1}{12}\delta^{2}l^{4}S_{1}E=0,$$

$$MB_{0}\beta c^{2}S_{0}^{3}-MB_{0}vc^{2}S_{0}^{2}+S_{0}Mc^{2}B_{0}-S_{0}l^{2}=0.$$

The following solutions are derived from solving this SAEs.


$$S_{0}=\frac{1}{3}\frac{v}{\beta }$$

$$S_{1}=\pm \frac{1}{6}\frac{\sqrt{\left(-12v^{2}+54\beta \right)}}{3\beta }\delta l$$

$$E=-\frac{12v^{2}}{l^{2}\left(-2v^{2}+9\beta \right)\delta^{2}}$$

$$c=\frac{3\sqrt{\beta }l}{\sqrt{MB_{0}\left(-2v^{2}+9\beta \right)}}$$

Case 1: (When E>0, i.e., and D=0)


$$U_{1,2}^{\pm }\left(x,t\right)=\frac{1}{3}\frac{v}{\beta }\pm \frac{v}{9\beta }\left(\sqrt{\frac{3qr\left(-12v^{2}+54\beta \right)}{\left(-2v^{2}+9\beta \right)}}\right){\text{sech}}_{qr}\left(\sqrt{E}\left(l\frac{x^{\gamma }}{\gamma }+c\frac{t^{\gamma }}{\gamma }\right)\right),$$

$$U_{3,4}^{\pm }\left(x,t\right)=\frac{1}{3}\frac{v}{\beta }\pm \frac{v}{9\beta }\left(\sqrt{-\frac{3qr\left(-12v^{2}+54\beta \right)}{\left(-2v^{2}+9\beta \right)}}\right){\text{csch}}_{qr}\left(\sqrt{E}\left(l\frac{x^{\gamma }}{\gamma }+c\frac{t^{\gamma }}{\gamma }\right)\right).$$

Case 2: (When E<0, i.e., and D=0)


$$U_{5,6}^{\pm }\left(x,t\right)=\frac{1}{3}\frac{v}{\beta }\pm \frac{v}{9\beta }\left(\sqrt{\frac{3qr\left(-12v^{2}+54\beta \right)}{\left(-2v^{2}+9\beta \right)}}\right)\sec_{qr}\left(\sqrt{-E}\left(l\frac{x^{\gamma }}{\gamma }+c\frac{t^{\gamma }}{\gamma }\right)\right),$$

$$U_{7,8}^{\pm }\left(x,t\right)=\frac{1}{3}\frac{v}{\beta }\pm \frac{v}{9\beta }\left(\sqrt{\frac{3qr\left(-12v^{2}+54\beta \right)}{\left(-2v^{2}+9\beta \right)}}\right)\csc_{qr}\left(\sqrt{-E}\left(l\frac{x^{\gamma }}{\gamma }+c\frac{t^{\gamma }}{\gamma }\right)\right).$$

Case 3: (When E<0, i.e., and $D=\frac{E^{2}}{4}$)


$$U_{9,10}^{\pm }\left(x,t\right)=\frac{1}{3}\frac{v}{\beta }\pm \frac{v}{18\beta }\left(\sqrt{\frac{6\left(-12v^{2}+54\beta \right)}{\left(-2v^{2}+9\beta \right)}}\right)\tanh_{qr}\left(\sqrt{-\frac{E}{2}}\left(l\frac{x^{\gamma }}{\gamma }+c\frac{t^{\gamma }}{\gamma }\right)\right),$$

$$U_{11,12}^{\pm }\left(x,t\right)=\frac{1}{3}\frac{v}{\beta }\pm \frac{v}{18\beta }\left(\sqrt{\frac{6\left(-12v^{2}+54\beta \right)}{\left(-2v^{2}+9\beta \right)}}\right)\coth_{qr}\left(\sqrt{-\frac{E}{2}}\left(l\frac{x^{\gamma }}{\gamma }+c\frac{t^{\gamma }}{\gamma }\right)\right).$$

$$\begin{array}{c} U_{13,14}^{\pm }\left(x,t\right)=\frac{1}{3}\frac{v}{\beta }\pm \frac{v}{18\beta }\left(\sqrt{\frac{6\left(-12v^{2}+54\beta \right)}{\left(-2v^{2}+9\beta \right)}}\right)(\tanh_{qr}\left(\sqrt{-2E}\left(l\frac{x^{\gamma }}{\gamma }+c\frac{t^{\gamma }}{\gamma }\right)\right)\pm i\sqrt{qr}\text{ sech}\left(\sqrt{-2E}\left(l\frac{x^{\gamma }}{\gamma }+c\frac{t^{\gamma }}{\gamma }\right)\right)), \end{array}$$

$$\begin{array}{c} U_{15,16}^{\pm }\left(x,t\right)=\frac{1}{3}\frac{v}{\beta }\pm \frac{v}{18\beta }\left(\sqrt{\frac{6\left(-12v^{2}+54\beta \right)}{\left(-2v^{2}+9\beta \right)}}\right)(\coth_{qr}\left(\sqrt{-2E}\left(l\frac{x^{\gamma }}{\gamma }+c\frac{t^{\gamma }}{\gamma }\right)\right)\pm \sqrt{qr}\;{\text{csch}}_{qr}\left(\sqrt{-2E}\left(l\frac{x^{\gamma }}{\gamma }+c\frac{t^{\gamma }}{\gamma }\right)\right)), \end{array}$$

$$U_{17,18}^{\pm }\left(x,t\right)=\frac{1}{3}\frac{v}{\beta }\pm \frac{v}{36\beta }\left(\sqrt{\frac{6\left(-12v^{2}+54\beta \right)}{\left(-2v^{2}+9\beta \right)}}\right)(\tanh_{qr}\left(\sqrt{-\frac{E}{8}}\left(l\frac{x^{\gamma }}{\gamma }+c\frac{t^{\gamma }}{\gamma }\right)\right)\pm \sqrt{qr}\coth_{qr}\left(\sqrt{-\frac{E}{8}}\left(l\frac{x^{\gamma }}{\gamma }+c\frac{t^{\gamma }}{\gamma }\right)\right)).$$

Case 4: (When E>0, i.e., and $D=\frac{E^{2}}{4}$)


$$U_{19,20}^{\pm }\left(x,t\right)=\frac{1}{3}\frac{v}{\beta }\pm \frac{v}{18\beta }\left(\sqrt{-\frac{6\left(-12v^{2}+54\beta \right)}{\left(-2v^{2}+9\beta \right)}}\right)\tan_{qr}\left(\sqrt{\frac{E}{2}}\left(l\frac{x^{\gamma }}{\gamma }+c\frac{t^{\gamma }}{\gamma }\right)\right),$$

$$U_{21,22}^{\pm }\left(x,t\right)=\frac{1}{3}\frac{v}{\beta }\pm \frac{v}{18\beta }\left(\sqrt{-\frac{6\left(-12v^{2}+54\beta \right)}{\left(-2v^{2}+9\beta \right)}}\right)\cot_{qr}\left(\sqrt{\frac{E}{2}}\left(l\frac{x^{\gamma }}{\gamma }+c\frac{t^{\gamma }}{\gamma }\right)\right),$$

$$U_{23,24}^{\pm }\left(x,t\right)=\frac{1}{3}\frac{v}{\beta }\pm \frac{v}{18\beta }\left(\sqrt{-\frac{6\left(-12v^{2}+54\beta \right)}{\left(-2v^{2}+9\beta \right)}}\right)(\tan_{qr}\left(\sqrt{2E}\left(l\frac{x^{\gamma }}{\gamma }+c\frac{t^{\gamma }}{\gamma }\right)\right)\pm \sqrt{qr}\sec\left(\sqrt{2E}\left(l\frac{x^{\gamma }}{\gamma }+c\frac{t^{\gamma }}{\gamma }\right)\right)),$$

$$U_{25,26}^{\pm }\left(x,t\right)=\frac{1}{3}\frac{v}{\beta }\pm \frac{v}{18\beta }\left(\sqrt{-\frac{6\left(-12v^{2}+54\beta \right)}{\left(-2v^{2}+9\beta \right)}}\right)(\cot_{qr}\left(\sqrt{2E}\left(l\frac{x^{\gamma }}{\gamma }+c\frac{t^{\gamma }}{\gamma }\right)\right)\pm \sqrt{qr}\csc_{qr}\left(\sqrt{2E}\left(l\frac{x^{\gamma }}{\gamma }+c\frac{t^{\gamma }}{\gamma }\right)\right)),$$

$$U_{27,28}^{\pm }\left(x,t\right)=\frac{1}{3}\frac{v}{\beta }\pm \frac{v}{36\beta }\left(\sqrt{-\frac{6\left(-12v^{2}+54\beta \right)}{\left(-2v^{2}+9\beta \right)}}\right)(\tan_{qr}\left(\sqrt{\frac{E}{8}}\left(l\frac{x^{\gamma }}{\gamma }+c\frac{t^{\gamma }}{\gamma }\right)\right)\pm \sqrt{qr}\cot_{qr}\left(\sqrt{\frac{E}{8}}\left(l\frac{x^{\gamma }}{\gamma }+c\frac{t^{\gamma }}{\gamma }\right)\right)).$$

## 4. Application of DSW equation in Sardar-subequation method

Within this part, the method outlined is used to determine novel wave solutions for the DSW equation, as specified in Eq. (1). In solving the equation we have presented, we consider wave transformation v(x,t)=V(ξ), u(x,t)=U(ξ) where $\xi =n(x-\delta \frac{t^{\beta }}{\beta })$, δ≠0 and relieving this transformation in Eq. (1), the resulting equation is imitative


$$\begin{array}{c} \left\{ \begin{array}{c} \;-n\delta U^{\prime }+anVV^{\prime }=0\\ -n\delta V^{\prime }+bnUU^{\prime }+cnVU^{\prime }+dn^{3}V^{\prime \prime \prime }=0. \end{array}\right. \end{array}$$ (9)

Based on the first equation in Eq. (9), we find


$$U=\frac{aV^{2}}{2\delta }.$$ (10)

Inserting (10) into the second equation from Eq. (9) and integrating leads to the ODE outlined below:


$$-6\delta^{2}V+a\left(b+2c\right)V^{3}+6d\delta n^{2}V^{\prime \prime }=0.$$ (11)

By balancing U'' and $U^{3},$ we get the value of N=1. From Eq. (5), we have


$$V\left(\xi \right)=S_{0}+S_{1}\mathrm{\backslash Upphi}(\xi )$$ (12)

Where $S_{0}andS_{1}$are actual arbitrary values which are unknowns and determined well ahead.

Substituting Eq. (12) and Eq. (6) into Eq. (11) yields a polynomial involving φ(ξ). Setting all the coefficients of φ(ξ) to zero provides the ensuing SAE:$abcS_{1}^{3}+2acS_{1}^{3}+12d\delta n^{2}S_{1}=0$


$$3abS_{0}S_{1}^{2}+6acS_{0}S_{1}^{2}=0$$

$$6Ed\delta n^{2}S_{1}+3abS_{0}^{2}S_{1}+6acS_{0}^{2}S_{1}-6\delta^{2}S_{1}=0$$

$$abS_{0}^{3}+2acS_{0}^{3}-6\delta^{2}S_{0}=0$$

The solution set derived from solving these SAEs is as follows:


$$S_{0}=0,S_{1}=\pm \frac{2\sqrt{-3a\left(b+2c\right)d\delta }n}{a\left(b+2c\right)},E=\frac{\delta }{dn^{2}}$$

Case 1: (When E>0, i.e., $\frac{\delta }{dn^{2}}>0$ and D=0)


$$V_{1,2}^{\pm }\left(x,t\right)=\pm \frac{4\sqrt{-\frac{qr\delta }{dn^{2}}}\sqrt{-3a\left(b+2cd\delta \right)}n}{a\left(b+2c\right)}{\text{sech}}_{qr}\left(\sqrt{E}\left(n\left(x-\delta \frac{t^{\beta }}{\beta }\right)\right)\right),$$

$$V_{3,4}^{\pm }\left(x,t\right)=\pm \frac{4\sqrt{\frac{qr\delta }{dn^{2}}}\sqrt{-3a\left(b+2cd\delta \right)}n}{a\left(b+2c\right)}{\text{sech}}_{qr}\left(\sqrt{E}\left(n\left(x-\delta \frac{t^{\beta }}{\beta }\right)\right)\right),$$

Case 2: (When E<0, i.e., $\frac{\delta }{dn^{2}}<0$ and D=0)


$$V_{5,6}^{\pm }\left(x,t\right)=\pm 4\frac{\sqrt{-\frac{qr\delta }{dn^{2}}}\sqrt{-3a\left(b+2cd\delta \right)}n}{a\left(b+2c\right)}\sec_{qr}\left(\sqrt{-E}\left(n\left(x-\delta \frac{t^{\beta }}{\beta }\right)\right)\right),$$

$$V_{7,8}^{\pm }\left(x,t\right)=\pm \frac{4\sqrt{-\frac{qr\delta }{dn^{2}}}\sqrt{-3a\left(b+2cd\delta \right)}n}{a\left(b+2c\right)}\csc_{qr}\left(\sqrt{-E}\left(n\left(x-\delta \frac{t^{\beta }}{\beta }\right)\right)\right),$$

Case 3: (When E<0, i.e., $\frac{\delta }{dn^{2}}<0$ and $D=\frac{E^{2}}{4}$)


$$V_{9,10}^{\pm }\left(x,t\right)=\pm \frac{\sqrt{-\frac{2\delta }{dn^{2}}}\sqrt{-3a\left(b+2cd\delta \right)}n}{a\left(b+2c\right)}\tanh_{qr}\left(\sqrt{-\frac{E}{2}}\left(n\left(x-\delta \frac{t^{\beta }}{\beta }\right)\right)\right),$$

$$V_{11,12}^{\pm }\left(x,t\right)=\pm \frac{\sqrt{-\frac{\delta }{2dn^{2}}}\sqrt{-3a\left(b+2cd\delta \right)}n}{a\left(b+2c\right)}\coth_{qr}\left(\sqrt{-\frac{E}{2}}\left(n\left(x-\delta \frac{t^{\beta }}{\beta }\right)\right)\right).$$

$$\begin{array}{c} V_{13,14}^{\pm }\left(x,t\right)=\pm \frac{\sqrt{-\frac{2\delta }{dn^{2}}}\sqrt{-3a\left(b+2cd\delta \right)}n}{a\left(b+2c\right)}(\tanh_{qr}\left(\sqrt{-2E}\left(n\left(x-\delta \frac{t^{\beta }}{\beta }\right)\right)\right)\pm i\sqrt{qr}\text{ sech}\left(\sqrt{-2E}\left(l\frac{x^{\gamma }}{\gamma }+c\frac{t^{\gamma }}{\gamma }\right)\right)), \end{array}$$

$$\begin{array}{c} V_{15,16}^{\pm }\left(x,t\right)=\pm \frac{\sqrt{-\frac{2\delta }{dn^{2}}}\sqrt{-3a\left(b+2cd\delta \right)}n}{a\left(b+2c\right)}(\coth_{qr}\left(\sqrt{-2E}\left(n\left(x-\delta \frac{t^{\beta }}{\beta }\right)\right)\right)\pm \sqrt{qr}\;{\text{csch}}_{qr}\left(\sqrt{-2E}\left(l\frac{x^{\gamma }}{\gamma }+c\frac{t^{\gamma }}{\gamma }\right)\right)), \end{array}$$

$$\begin{array}{c} U_{17,18}^{\pm }\left(x,t\right)=\pm \frac{\sqrt{-\frac{2\delta }{dn^{2}}}\sqrt{-3a\left(b+2cd\delta \right)}n}{2a\left(b+2c\right)}(\tanh_{qr}\left(\sqrt{-\frac{E}{8}}\left(n\left(x-\delta \frac{t^{\beta }}{\beta }\right)\right)\right)\pm \sqrt{qr}\;\coth_{qr}\left(\sqrt{-\frac{E}{8}}\left(n\left(x-\delta \frac{t^{\beta }}{\beta }\right)\right)\right)). \end{array}$$

Case 4: (When E>0, i.e., $\frac{\delta }{dn^{2}}>0$ and $D=\frac{E^{2}}{4}$)


$$V_{19,20}^{\pm }\left(x,t\right)=\pm \frac{\sqrt{2}\sqrt{\frac{\delta }{dn^{2}}}\sqrt{-3a\left(b+2cd\delta \right)}n}{a\left(b+2c\right)}\tan_{qr}\left(\sqrt{\frac{E}{2}}\left(n\left(x-\delta \frac{t^{\beta }}{\beta }\right)\right)\right),$$

$$V_{21,22}^{\pm }\left(x,t\right)=\pm \frac{\sqrt{2}\sqrt{\frac{\delta }{dn^{2}}}\sqrt{-3a\left(b+2cd\delta \right)}n}{a\left(b+2c\right)}\cot_{qr}\left(\sqrt{\frac{E}{2}}\left(n\left(x-\delta \frac{t^{\beta }}{\beta }\right)\right)\right),$$

$$V_{23,24}^{\pm }\left(x,t\right)=\pm \frac{\sqrt{2}\sqrt{\frac{\delta }{dn^{2}}}\sqrt{-3a\left(b+2cd\delta \right)}n}{a\left(b+2c\right)}(\tan_{qr}\left(\sqrt{2E}\left(l\frac{x^{\gamma }}{\gamma }+c\frac{t^{\gamma }}{\gamma }\right)\right)\pm \sqrt{qr}\sec\left(\sqrt{2E}\left(n\left(x-\delta \frac{t^{\beta }}{\beta }\right)\right)\right)),$$

$$V_{25,26}^{\pm }\left(x,t\right)=\pm \frac{\sqrt{2}\sqrt{\frac{\delta }{dn^{2}}}\sqrt{-3a\left(b+2cd\delta \right)}n}{a\left(b+2c\right)}(\cot_{qr}\left(\sqrt{2E}\left(n\left(x-\delta \frac{t^{\beta }}{\beta }\right)\right)\right)\pm \sqrt{qr}\csc_{qr}\left(\sqrt{2E}\left(n\left(x-\delta \frac{t^{\beta }}{\beta }\right)\right)\right)),$$

$$V_{27,28}^{\pm }\left(x,t\right)=\pm \frac{\frac{\sqrt{2}}{4}\sqrt{\frac{\delta }{dn^{2}}}\sqrt{-3a\left(b+2cd\delta \right)}n}{a\left(b+2c\right)}(\tan_{qr}\left(\sqrt{\frac{E}{8}}\left(n\left(x-\delta \frac{t^{\beta }}{\beta }\right)\right)\right)\pm \sqrt{qr}\cot_{qr}\left(\sqrt{\frac{E}{8}}\left(n\left(x-\delta \frac{t^{\beta }}{\beta }\right)\right)\right)).$$

## 5. Results and discussions

**Fig 1:**
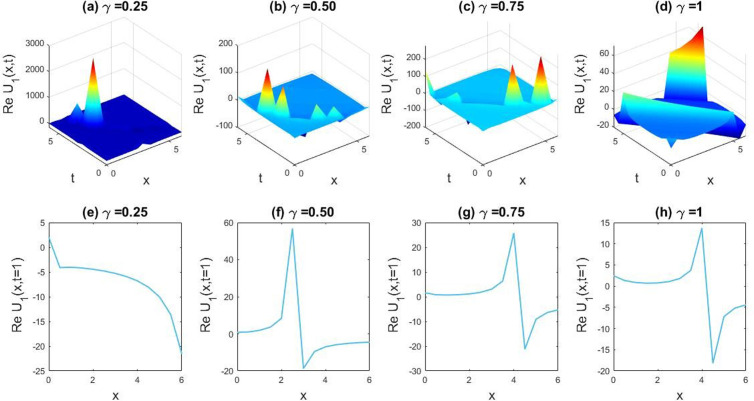
Showing the dynamical diversity emerging from the obtained solution of $\begin{array}{c} U_{1}(x,t) \end{array}$ with parametric values $\begin{array}{c} 𝐥=.5,\;𝐯=-0.05,\;\delta =1,\;\beta =0.01,\;q=1,\;r=1,\;B_{0}=1,\;M=1. \end{array}$ Subfigures $\begin{array}{c} (a,b,c,d) \end{array}$ provide 3D plot views. Additionally, sub figures $\begin{array}{c} \left(e,f,g,h\right) \end{array}$ represent the 2D line charts for y=0 in accordance with the particular figures $\begin{array}{c} \left(a,b,c,d\right) \end{array}$.

**Fig 2:**
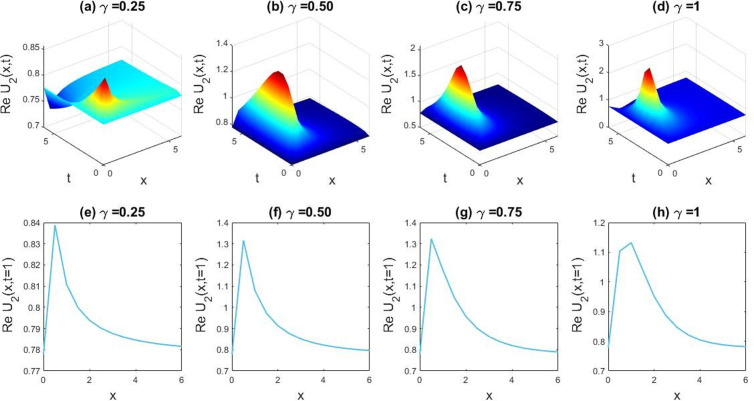
Showing the dynamical diversity emerging from the obtained solution of $\begin{array}{c} U_{2}\left(x,t\right) \end{array}$ with parametric values $\begin{array}{c} l=-0.5,v=7,\delta =3,\beta =3,q=1,r=1,B_{0}=1,M=1. \end{array}$ Subfigures $\begin{array}{c} \left(a,b,c,d\right) \end{array}$ provide 3D plot views. Additionally, sub figures (e,f,g,h) represent the 2D stripe diagrams for y=0 in accordance with the particular diagrams $\begin{array}{c} \left(a,b,c,d\right) \end{array}$**.**

**Fig 3:**
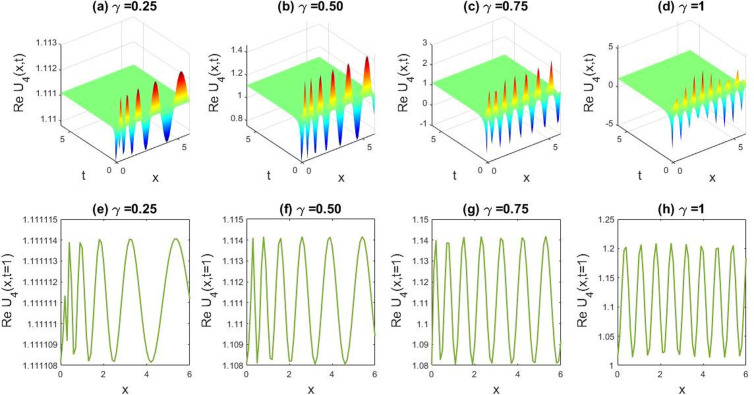
Demonstrating the dynamical diversity emerging from the obtained solution of $\begin{array}{c} U_{4}(x,t) \end{array}$ with the preferred values, $\begin{array}{c} l=-0.9,v=10,\delta =0.3,\beta =3,q=1,r=1,B_{0}=1,M=1. \end{array}$ Subfigures (a,b,c,d) provide 3D plot views. Additionally, sub figures (e,f,g,h) represent the 2D line charts for y=0 conforming to particular charts $\begin{array}{c} \left(a,b,c,d\right) \end{array}$.

**Fig 4:**
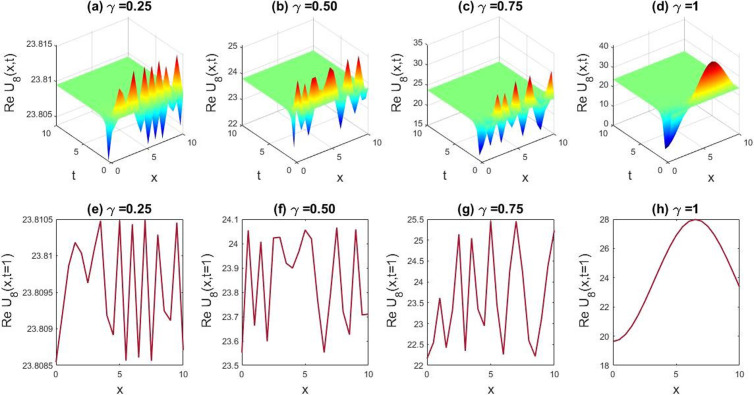
Describing the dynamical diversity emerging from the obtained solution of $\begin{array}{c} U_{8}(x,t) \end{array}$ with parametric values, $\begin{array}{c} l=0.5,v=5,\delta =0.1;\beta =0.07,q=1,r=1,B_{0}=1,M=1,t=1. \end{array}$ Subfigures (a,b,c,d) provide 3D plot views. Additionally, sub figures (e,f,g,h) represent the 2D line charts for y=0 and t = 1 conforming to particular charts $\begin{array}{c} \left(a,b,c,d\right) \end{array}$.

**Fig 5:**
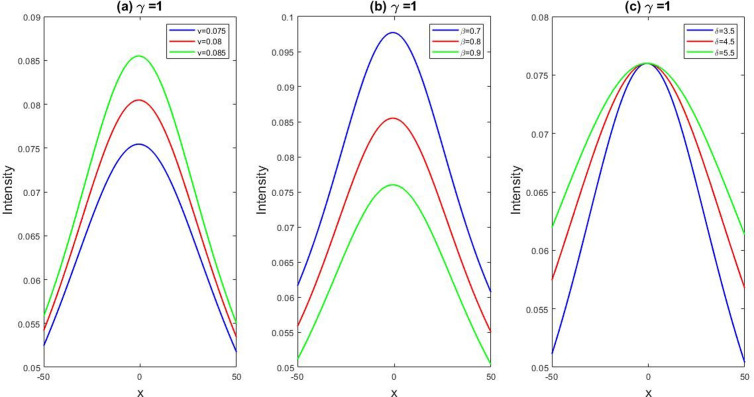
Depicting the dynamic behaviors of $\begin{array}{c} U_{1}(x,t) \end{array}$ for the parametric values, $\begin{array}{c} l=1.4,M=1,q=1,r=1,\gamma =1,B_{0}=1 \end{array}$ and the graphical variation of $\begin{array}{c} U_{1}(x,t) \end{array}$ for the changes of free variables $\begin{array}{c} v=0.075\;to\;0.085,\beta =0.7\;to\;0.9,\;and\;\;\delta =3.5 \end{array}$ to 5.5.

**Fig 6:**
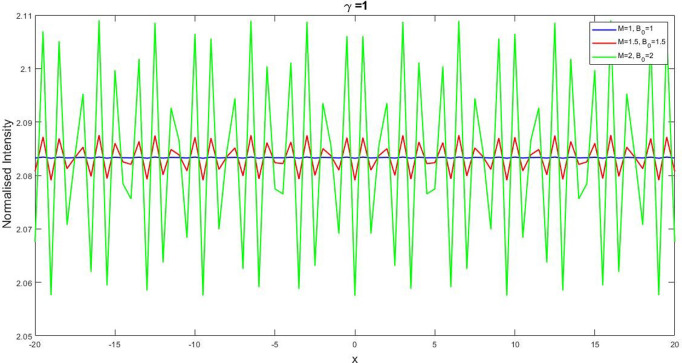
Describing the solution graph of $\begin{array}{c} U_{1}(x,t) \end{array}$ for the parameters $\begin{array}{c} l=0.11,\beta =0.5,v=5,\delta =0.5,\beta =0.8,q=1,and\;r=1 \end{array}$ and the graphical variation of $\begin{array}{c} U_{1}(x,t) \end{array}$ for the changes of free variables $M=1\text{ to }2,\text{and }B_{0}=1\text{ to }2$.

**Fig 7:**
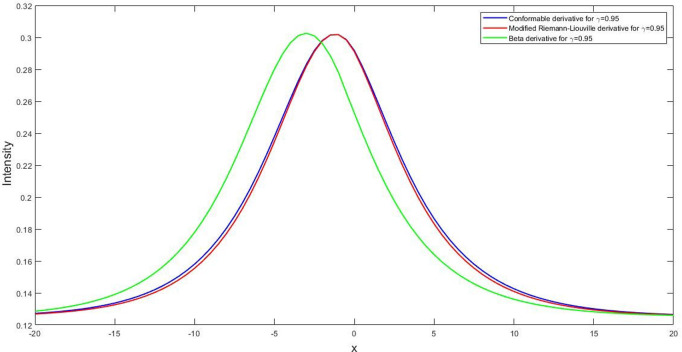
System of 2D curves of $\begin{array}{c} U_{1}(x,t) \end{array}$ for the parametric values $\begin{array}{c} l=1.5v=3.\delta =5,\beta =8,q=1,r=1,t=1,M=1,B_{0}=1 \end{array}$ and γ=0.95 with the sense of conformable derivative, modified Riemann-Liouville derivative, and beta derivative respectively.

**Fig 8:**
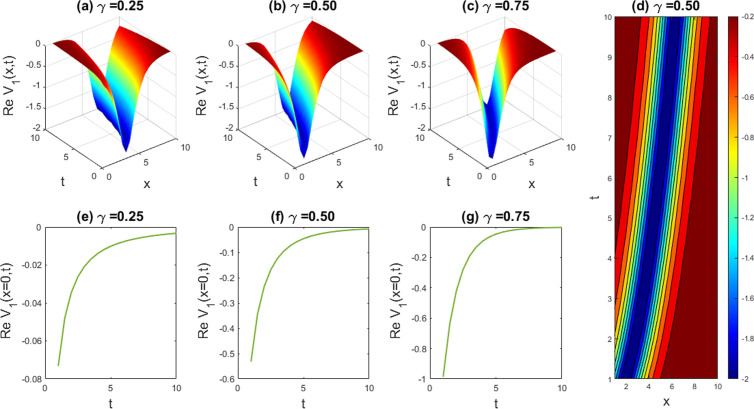
Exploring the dynamic performance arising from the attained solution of $\begin{array}{c} {\left|V_{1}(x,t)\right|}^{2} \end{array}$ with parameters $\begin{array}{c} a=1,b=1,c=1,d=1,q=1,r=1,n=1, \end{array}$ and δ=1. Subfigures (a,b,c) offer 3D perspectives, (d) represents the contour intrigue, and (e,f,g) exhibits 2D line charts at x=0 consistent to the related 3D visions.

**Fig 9:**
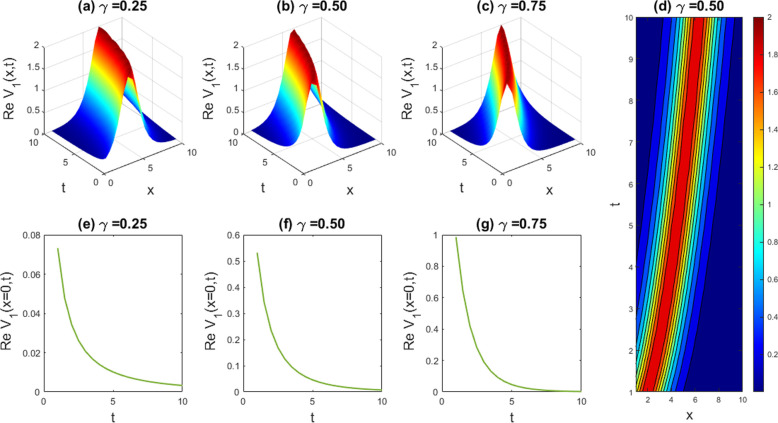
Exploring the dynamic comportment ascending taken from the attained solution of  $\begin{array}{c} {\left|V_{1}(x,t)\right|}^{2} \end{array}$ with parameters $\begin{array}{c} a=1,b=1,c=1,d=1,q=1,r=1,n=-1, \end{array}$ and $\begin{array}{c} \delta =1 \end{array}$. Subfigures (a,b,c) offer 3D perspectives, (d) represents the contour intrigue, and (e,f,g) exhibits 2D line charts at x=0 equivalent to the related 3D visions.

**Fig 10:**
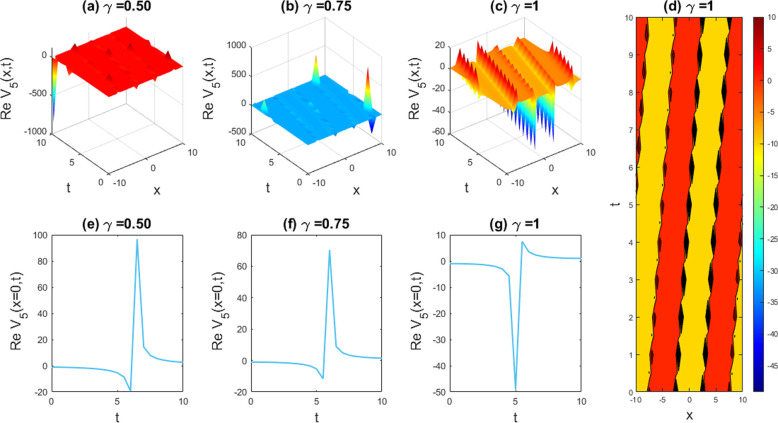
Exploring the vibrant diversity ascending from the attained solution of $\begin{array}{c} {\left|V_{5}(x,t)\right|}^{2} \end{array}$ with parameters $\begin{array}{c} a=1,b=1,c=1,d=-1.3,q=1,r=1,n=1.2, \end{array}$ and δ=1. Subfigures (a,b,c) offer 3D perspectives, (d) represents the contour intrigue, and (e,f,g) exhibits 2D line charts at x=0 consistent to the related 3D sights.

**Fig 11:**
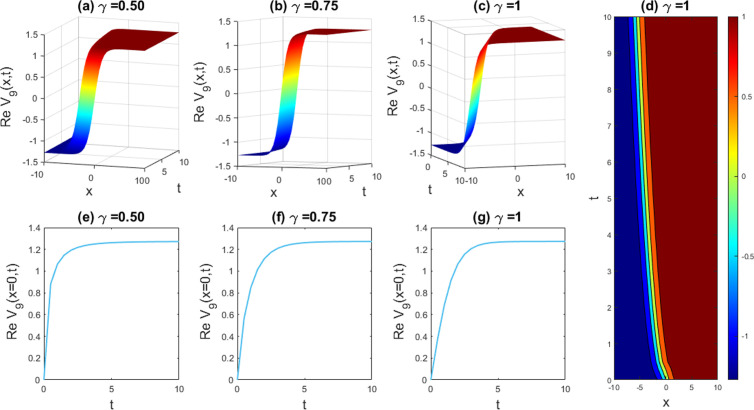
Exploring the lively comportment ascending from the attained solution of $\begin{array}{c} {\left|V_{9}(x,t)\right|}^{2} \end{array}$ with parameters $\begin{array}{c} a=1,b=1,c=1,d=-1,q=1,r=1,n=1.4, \end{array}$ and δ=1.4. Subfigures (a,b,c) offer 3D perspectives, (d) represents the contour design, and (e,f,g) exhibits the 2D line charts at x=0 equivalent to the related 3D visions.

Graphical examples of different solutions are given in this part to underscore their physical significance. The **Sardar-subequation** technique is utilized to extract some new waveform to the LPET line equation and DSW model. It is appropriate to assert here, based on the obtained solutions, that the employed technique can proficiently solve the problem of our attention with the help of symbolic computational software Maple-17, which can effortlessly address the involved and time consuming calculations and greatly decrease computation time. The obtained singular and multiple soliton, kink and periodic kink, W-shaped bright soliton, dark-kink soliton and kink-like soliton solutions, as well as their physical descriptions, are graphically depicted with the free parameters (such as the nonlinearity feature, dispersion feature, etc.) set to the values indicated above. The graphical outcomes of the solutions mentioned are shown in Figs 1–11. In the next subsections, the discussion includes the implications of fractionality and the influence of the free parameters in the model equation as demonstrated by the various solutions obtained.

### 5.1. Graphical representation of Space-time fractional LPET model

**Fig 12:**
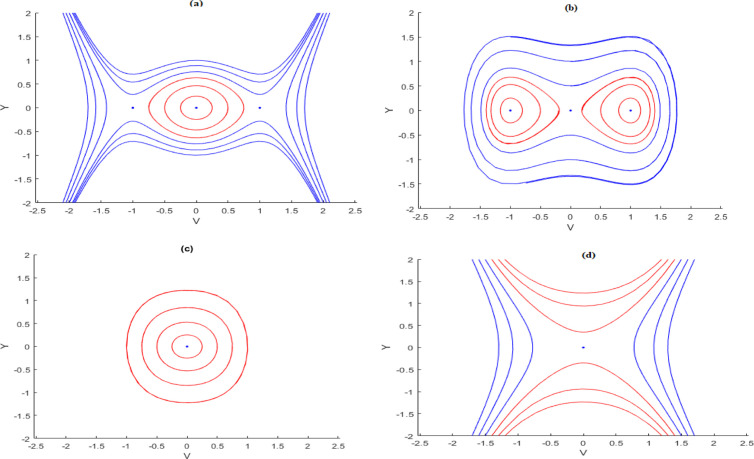
Visualization of phase diagrams of the dynamical system Eq. (23).

In this section we have gathered some graphical results of attained solutions of our another governing LPET equation. The possessions of fractionality are scrutinized through several solutions of the space-time fractional LPET line equation, with Figs 1–4 providing insights into this and Figs 9–12 offering a better perspective on the DSW equation. In Fig 1, the soliton result is depicted in both three dimensional (3D) and two dimensional (2D) plots to demonstrate the effect of fractionality of assuming the appropriate parameters $l=0.5,v=-0.05,\delta =1,\beta =0.01,q=1,r=1,B_{0}=1,\text{ and}\;M=1.$ By varying the fractional parameter γ=0.25 to 1, the multiple soliton form deviations to singular soliton shape. In Fig 2, we exhibited the kink wave soliton solution of Real part of for the parameters $l=-0.5,v=7,\delta =3,\beta =3,q=1,r=1,B_{0}=1,M=1$. For changing the fractional parameter γ=0.25 to 1 the frequency of the soliton shape has been slightly changed in shape. In Fig 3, we found the periodic kink soliton solution of Real part of for the parameters $l=-0.9,v=10,\delta =0.3,\beta =3,q=1,r=1,B_{0}=1,M=1$. For variation of the fractional parameter γ=0.25 to 1 the periodic kink soliton shape has no dynamical changes in its amplitude and frequency. In Fig 4, we found the periodic kink soliton solution of Real part of for the parameters $l=0.5,v=5,\delta =0.1;\beta =0.07,q=1,r=1,B_{0}=1,M=1,t=1$. For variation of the fractional parameter γ=0.25 to 1 the periodic kink soliton shape changes to the singular kink wave. Rest of the obtained solutions have almost the same physical phenomena. For this reason, we have skipped the coincide one.

#### 5.1.1. Effects of free parameters with fixed fractionality of LPET equation.

In this subsection, we have discussed the belongings of unrestricted parameters of the obtained solution $U_{1}(x,t)$ derived from. Here, we have fixed the fractional order derivatives γ=1. We have depicted the Fig 5 for the parameters $l=1.4,M=1,q=1,r=1,\gamma =1,B_{0}=1$ of $U_{1}(x,t)$ and we also showed the graphical variation of $U_{1}(x,t)$ for the changes of free variables v=0.075 to 0.085,β=0.7 to 0.9, andδ=3.5 to 5.5. In Fig 5(a), we observed that if we keep the parameters l=1.4,M=1,q=1,r=1,γ=1,and $B_{0}=1$ fixed and the free variables v changes 0.075 to 0.085 then the frequency of the wave amplitude will increase. In Fig 5(b), we observed that if we keep the mentioned parameters fixed and the free variables βchanges 0.7 to 0.9 then the intensity of the wave amplitude will decrease. In Fig 5(c), we observed that if we keep the mentioned parameters fixed and the free variables δchanges 3.5 to 5.5 then the wave amplitudes will coincide in a fixed critical point and after that the frequency will spread out with the increases of δ. We have depicted the Fig 6 for the values, l=0.11,β=0.5,v=5,δ=0.5,β=0.8,q=1,r=1 of $U_{1}\left(x,t\right)$ and we also showed the graphical variation of $U_{1}(x,t)$ for the changes of free variables M=1 to 2 and $B_{0}=1$to 2. Here, our finding is that, when we put the value M=1 and $B_{0}=1$in $U_{1}(x,t)$ the frequency of amplitude is very low. When we increase this value then the frequency of the amplitude is relatively high. So we can conclude that our obtained solution is highly sensitive with ever small changes of its fractional and free parameters.

#### 5.1.2. Comparative study with different fractional derivatives.

The solutions from fractional model give unique physical perspectives and submissions by taking into account the fractional values of these FDs, given the assorted definitions of FDs like conformable, modified Riemann-Liouville [62], and beta derivative [63]. It is challenging for scientists to identify the most accurate FD. However, the following discussion contrasts the solutions acquired in this part with those derived from the modified Riemann-Liouville and the beta derivatives. Here we have the two traveling wave transformation in form of modified Riemann-Liouville derivative $\xi =\frac{lx^{\gamma }}{\mathrm{\backslash Upgamma}(1+\gamma )}+\frac{ct^{\gamma }}{\mathrm{\backslash Upgamma}(1+\gamma )}$ and the beta derivative $\xi =\frac{l}{\gamma }{\left(x+\frac{1}{\mathrm{\backslash Upgamma}\left(\gamma \right)}\right)}^{\gamma }+\frac{c}{\gamma }{\left(t+\frac{1}{\mathrm{\backslash Upgamma}\left(\gamma \right)}\right)}^{\gamma }.$ With the help of our prescribed Sardar-subequation method, we imposed these wave transformations on our governing LPTE equations. As a consequence, we got the analytic solutions that we showed graphically with the assists of some free parameters. Our findings in this phenomena are that, forγ=0.95 the conformable derivative and modified Riemann-Liouville derivative gives us the almost same frequency and amplitude of equation $U_{1}(x,t)$ but for beta derivatives the nature of the intensity is slightly changed in its shape and size.

### 5.2. Graphical illustration of the DSW equations

In this segment, we have gathered approximately graphical results of obtained solutions of our another governing DSW equation. Fig 8 demonstrates the effects of various unknown parameters and fractionality, visualized through 3D, 2D, and density plots of W-shaped dark-kink solution of assuming the appropriate parameters a=1,b=1,c=1,d=1,q=1,r=1,n=1,δ=1. With the alteration of fractional parameter γ=0.25 to 1, the W-shaped soliton shape has no changes to its frequency and amplitude. In Fig 9, we displayed the bright-kink singular soliton solution of Real part of for the parameters a=1,b=1,c=1,d=1,q=1,r=1,n=−1,and δ=1. For changing the fractional parameter γ=0.25 to 1, the frequency of the soliton form has no changes in its shape. In Fig 10, we disclosed the singular type soliton solution of real part of for the parameters a=1,b=1,c=1,d=−1.3,q=1,r=1,n=1.2,and δ=1. For changing the fractional parameter γ=0.25 to 1, the frequency of the soliton shape change to multiple soliton solution shape. In Fig 11, we showed the kink-like soliton solution of Real part of for the a=1,b=1,c=1,d=−1,q=1,r=1,n=1.4,and δ=1.4. For changing the fractional parameter γ=0.25 to 1, the frequency of the soliton shape has no changes in its shape. Rest of the obtained solutions have almost the same physical phenomena. For this reason, we have skipped the coincide one.

## 6. Bifurcation analysis

This part examines the bifurcation and phase portraits of a planar dynamical system. By employing dynamical system methods, one can qualitatively analyze nonlinear partial differential models [64]. The systems orbits may apparent as points, simple closed arcs, or further shapes, each corresponding to different physical solutions of Eq. (11). By setting $\frac{dV}{d\xi }=Y$, the planar dynamical form of Eq. (11) in terms of a Hamiltonian function, which can be written as [65,66]:


$$\frac{dV}{d\xi }=Y,\frac{dY}{d\xi }=-gV-hV^{3},$$ (13)

$$H\left(V,Y\right)=\frac{Y^{2}}{2}+\frac{gV^{2}}{2}+\frac{hV^{4}}{4}=h$$ (14)

Where $g=-\frac{\delta }{dn^{2}},h=\frac{a\left(b+2c\right)}{6d\delta n^{2}}$ and h is the Hamiltonian constant.

To find the equilibrium points of the system described by Eq. (13), we need to solve the system of equations $Y=0,-gV-hV^{3}=0$. In this case, solving the system of equations yields a single equilibrium point at (0,0), when gh>0. On the other hand, when gh<0, three equilibrium points are found: $\left(0,0\right),\left(\sqrt{-\frac{g}{h}},0\right)$and $\left(-\sqrt{-\frac{g}{h}},0\right)$.

The Jacobian matrix for the system described by Eq. (13) has a determinant in the following form:


$$J\left(V,Y\right)=\left|\begin{array}{cc} 0 & 1\\ -3hV^{2}-g & 0 \end{array}\right|=3hV^{2}+g.$$ (15)

Thus, Eq. (15) yields the characteristic value $\sqrt{-3hV^{2}-g}$ at the position (V,0).As an outcome, the equilibrium point (V,0)behaves as a center point when the determinant J(V,Y) is positive, as a saddle point when J(V,Y) is negative, and as a cuspid-al point when J(V,Y)=0. Different parameters in the system (23) can attain in the following possible outcomes:

Case 1: h>0,g<0

By selecting the parameter set with a=−2,b=1,c=1,d=1,δ=1 and n=1,the system reveals three equilibrium points: (0,0),(1,0) and (−1,0) as depicted in Fig 12(a). The point (0,0) serves as a center, while (1,0) and (−1,0) act as saddle points. Fig 12(a) further shows that anti-kink and kink wave patterns emerge from the connection of the hetero clinic orbits at (1,0) and (−1,0).

Case 2: h<0,g>0

By taking the parameter set a=−2,b=1,c=1,d=1,δ=−1 and n=1, three equilibrium points are identified: (0,0),(1,0), and (−1,0), as shown in Fig 12(b). In this case, (0,0) acts as a saddle point, while (1,0) and (−1,0) are center points. The trajectories form closed curves, illustrating various solutions such as hyper-periodic (blue curve) and periodic (red curve).

Case 3: h>0,g>0

By picking the parameter set a=2,b=1,c=1,d=1,δ=−1 and n=1, the system identifies a single equilibrium point at (0,0)as shown in Fig 12(c). In this case,(0,0) is a center point. Fig 12(c) displays one family of periodic orbits that can be resulting from Eq. (13).

Case 4: h<0,g<0

By setting the parameters to a=2,b=1,c=1,d=1,δ=1 and n=1, only one equilibrium point, (0,0) is recognized, as shown in Fig 12(d). In this case, (0,0) acts as a saddle point. Notably, there are no closed trajectories in the system described by Eq. (13).

## 7. Chaotic behaviors

The chaotic behavior of the ensuing dynamical system is examined in this section through the consideration of perturbed terms and the analysis of 2D and 3D phase portraits. To start, we look at the resulting dynamical system:


$$\frac{dV}{d\xi }=Y,\frac{dY}{d\xi }=-gV-hV^{3}+\sigma \cos(\omega \xi )$$ (16)

**Fig 13:**
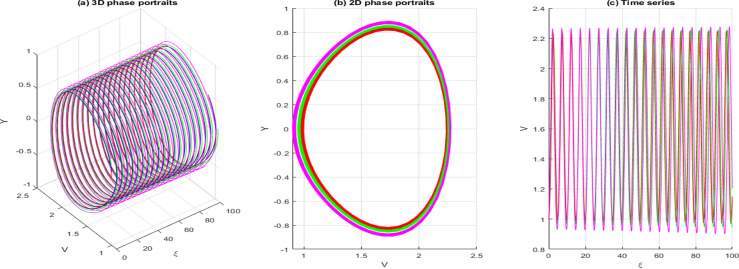
Behavior of Eq. (16) showing periodicity when $\begin{array}{c} \sigma =0 \end{array}$.

**Fig 14:**
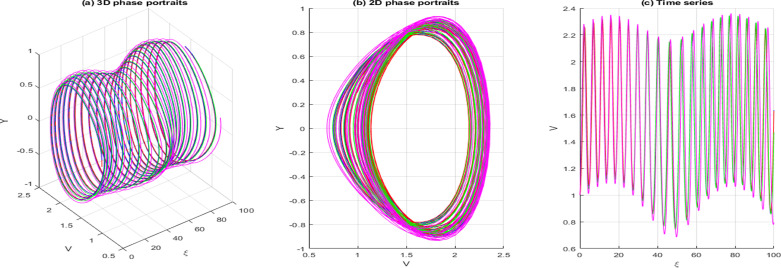
Behavior of Eq. (16) showing quasi-periodicity $\begin{array}{c} \sigma =0.1\;\omega =0.1 \end{array}$.

**Fig 15:**
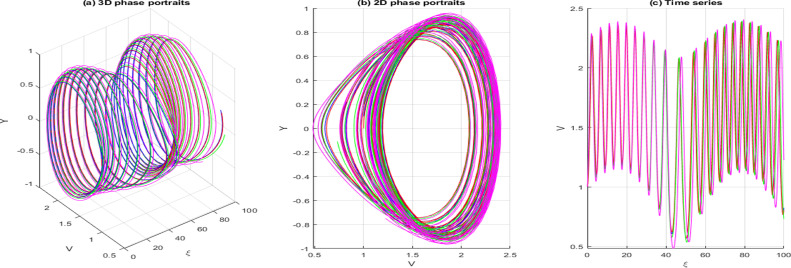
Behavior of Eq. (16) showing quasi-periodicity when $\begin{array}{c} \sigma =0.3\;\omega =0.1 \end{array}$.

**Fig 16:**
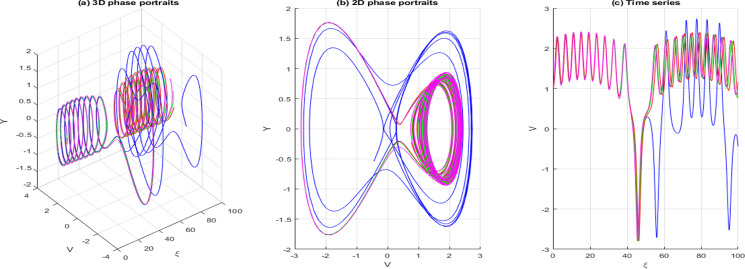
Behavior of Eq. (15) showing chaotic when $\begin{array}{c} \sigma =0.4,\omega =0.3 \end{array}$.

In this context, σcos(ωξ) signifies the perturbed term, where σdenotes the amplitude, and ωis the system’s frequency. In this section, we investigate the effects of perturbation intensity and frequency on the system described by Eq. (16). By holding the main parameters constant to a=−2,b=1,c=−1,d=1,δ=1 and n=1, we observe quasi-periodic and chaotic behaviors at various perturbation strengths and frequencies, as illustrated in Figs 13–16. Fig 13 illustrates the behavior of Eq. (16) whenσ=0, highlighting the system’s trajectory based on perturbation strength and frequency. In this case, the system exhibits periodic behavior, as shown in the time series, 2D, and 3D phase projections. In contrast, the results in Figs 14 and 15 demonstrate that a slight increase in perturbation strength and frequency (σ increases to 0.3 and ω=0.1)shifts the system from a periodic to a quasi-periodic state. Finally, in Fig 16, with a significant increase in both strength and frequency (σ increases to 0.4 and ω=0.3), the system experiences intense disturbances, leading to chaotic behavior.

## 8. Sensitivity analysis

**Fig 17:**
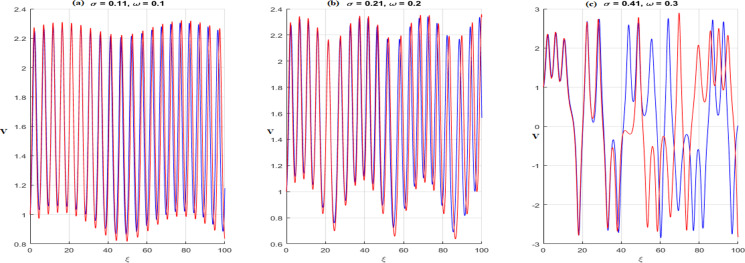
Sensitivity of Eq. (16) with initial conditions ($\begin{array}{c} 1,0.1 \end{array}$) (blue curve) and (1,0.2) (red curve).

This section examines in what way initial values touch the perturbed system defined by Eq. (16) across numerous strengths and frequencies, whereas keeping the parameters constant (a=−2,b=1,c=−1,d=1,δ=1 and n=1). Fig 17 displays the results, with a blue curve demonstrating a time series plot for initial values (V(0),Y(0))=(1,0.1) and a red curve for (V(0),Y(0))=(1,0.3). In Fig 17(a), the quasi-periodic nature of the system is evident for σ=0.11 and ω=0.1. Fig 17(b) shows that with a minor perturbation strength (σ=0.21 and ω=0.2), the time series plots exhibit minimal differences, indicating low sensitivity to initial conditions. However, in Fig 17(c), with increased perturbation strength (σ=0.41 and ω=0.3), significant variations between the time series plots are observed, highlighting a greater sensitivity to initial values.

## Conclusion

We have commendably explored bifurcation analysis, chaotic behaviors, and sensitivity analysis of DSW equation and explicit waveforms to the LPET and DSW equation that appeared in shallow water waves. By applying the Galilean conversion, we have effectively attained the dynamical structure of the stated equations, simplifying an inclusive bifurcation analysis. Furthermore, we explored several solitary wave solutions, including singular and multiple soliton, kink and periodic kink, W-shaped bright soliton, dark-kink soliton and kink-like soliton waves. We emphasized the unique features and presence of these solutions through the use of simulations to graphically show them. The outcomes demonstrate the efficacy, succinctness, and effectiveness of the integration methods used. They also imply that they may be used to explore more complex nonlinear models that are appearing in contemporary engineering and science contexts.
